# Iversion of Uterus in a Mare

**Published:** 1880-10

**Authors:** 


INVERSION OF UTERUS IN A MARE.—During my vacation of the present summer, I was called to see a mare which was said "to be sick from foaling"

On visiting the animal I found her in a condition demanding immediate relief. She had foaled the night before, or about fourteen hours previous.

Efforts had been made by the owner and assistants to pull out the mass, and finally some one had suggested pushing it back, and attempts had been made to do so. The whole of the uterus was inverted and considerably bruised and lacerated. The patient was very weak and scarcely able to stand. She was breathing rapidly; pulse was small, weak, and rapid; eyes sunken, and her general appearance one of collapse.

I at once attempted to reduce the inversion.

I first ordered an attendant to thoroughly cleanse the part with quite warm water.

Then, having thoroughly oiled the hands of my assistants with a weak solution of oil and carbolic acid, I directed them to carefully support and steady the uterus while I, putting my hand in the centre of the mass, began pushing it in the direction of the long axis of the body to the extent of about an arm's length. It was maintained in this position by one of my assistants, who followed up my hand with his Withdrawing my own hand, I began pushing and working in portions, and in a few minutes the whole mass was returned. The mare strained violently, and we had difficulty in preventing her from again expelling the uterus. This was accomplished by the attendant keeping his arm in the uterus until the expulsive pains were controlled by hypodermic injections of morphine and chloral hydrate.

As soon as the straining ceased in a measure and the patient's strength had been restored by doses of whiskey and warm gruel, I ordered the hindquarters to be raised and a watch kept on the animal.

On visiting my patient the next day, I found her a little feverish, off feed, pulse about 50, respiration slightly accelerated, no abdominal tenderness.

The next day the above symptoms subsided, and the animal has since appeared as usual.

I gave no internal medication, except drachm doses of fluid extract of gelsemium, every two hours.

